# Discovery of potent and noncovalent KRAS^G12D^ inhibitors: Structure-based virtual screening and biological evaluation

**DOI:** 10.3389/fphar.2022.1094887

**Published:** 2022-12-22

**Authors:** Yuting Wang, Hai Zhang, Jindong Li, Miao-Miao Niu, Yang Zhou, Yuanqian Qu

**Affiliations:** ^1^ Department of Pharmaceutical Analysis, China Pharmaceutical University, Nanjing, China; ^2^ Department of Hepatobiliary Surgery, The Affiliated Hospital of Jiangsu University, Zhenjiang, China; ^3^ Institute of Clinical Medicine, Department of Pharmacy, The Affiliated Taizhou People’s Hospital of Nanjing Medical University, Taizhou, China; ^4^ Department of Pathology, Department of Gastrointestinal Surgery, The Affiliated Changzhou Second People’s Hospital of Nanjing Medical University, Changzhou, China

**Keywords:** KRAS^G12D^, pancreatic cancer, inhibitor, virtual screening, biological evaluation

## Abstract

KRAS^G12D^, the most common oncogenic KRAS mutation, is a promising target for the treatment of pancreatic cancer. Herein, we identified four potent and noncovalent KRAS^G12D^ inhibitors (hits 1–4) by using structure-based virtual screening and biological evaluation. The *in vitro* assays indicated that the four compounds had sub-nanomolar affinities for KRAS^G12D^ and showed a dose-dependent inhibitory effect on human pancreatic cancer cells. In particular, the hit compound 3 was the most promising candidate and significantly inhibited the tumor growth of pancreatic cancer in tumor-bearing mice. The hit compound 3 represented a promising starting point for structural optimization in hit-to-lead development. This study shows that hit compound 3 provides a basis for the development of the treatment of cancer driven by KRAS^G12D^.

## 1 Introduction

Pancreatic cancer is one of the most invasive diseases with almost the same mortality in many countries (Elsayed and Abdelrahim, 2021; Sung et al., 2021). It has the lowest 5-year survival rate of 7% among cancer types (Ducreux et al., 2015; Zhou et al., 2018). The low early diagnosis rate and strong therapeutic resistance largely limit the effective treatment of patients with pancreatic cancer. Most of the patients were already in the unresectable stage at the time of diagnosis. In addition, the tumor is resistant to all forms of routine clinical treatment. It is estimated that pancreatic cancer will become the second leading cause of cancer-related deaths by 2030 (Bazhin et al., 2014; Busato et al., 2022), which highlights the need to develop new treatment drugs to improve the survival rate of patients with pancreatic cancer.

KRAS (Kirsten Ras protein) was found to be the most common locus for somatic gain-of-function mutations in patients with pancreatic cancer, accounting for about 95% (Morris et al., 2010; Eibl and Rozengurt, 2019). Recent studies have revealed that mutated KRAS expression is associated with reduced anticancer activity of gemcitabine and paclitaxel, the drugs currently used in the clinical treatment of pancreatic cancer, and new therapeutic strategies of targeting KRAS should inhibit tumor cell growth and combat drug resistance (Kimmelman, 2015; Grasso et al., 2017). The majority of cancer-associated hotspot mutations occur at codons G12, G13, and Q61, wherein G12 represents the most common mutation site (Kargbo, 2022). Among dominant mutations at G12, G12D has the highest mutation frequency (35.4%), followed by G12V (23.5%), G12R (8.7%) and G12C (4.3%), which has aroused the interest of many drug researchers in developing new cancer treatment methods targeting KRAS^G12D^ mutant (Salem et al., 2021). Mutations in RASA, the central signal transduction molecule, directly impair the intrinsic GTPase activity of KRAS and prevent the GTPase activating protein (GAP) from promoting the conversion of active GTP to inactive GDP (Wang et al., 2021). KRAS proteins then bind to GTP and activate downstream effector proteins and signaling pathways (mechanistic targets of mitogen-activated protein kinase (MAPK) -MAPK kinase (MEK) and phosphatidylinositol 3-kinase (PI3K) -Akt-rapamycin (mTOR)), leading to sustained cell proliferation (Buscail et al., 2020; Zheng et al., 2022). Therefore, targeting KRAS^G12D^ has the potential to block the occurrence and development of pancreatic cancer from the source.

**FIGURE 1:**
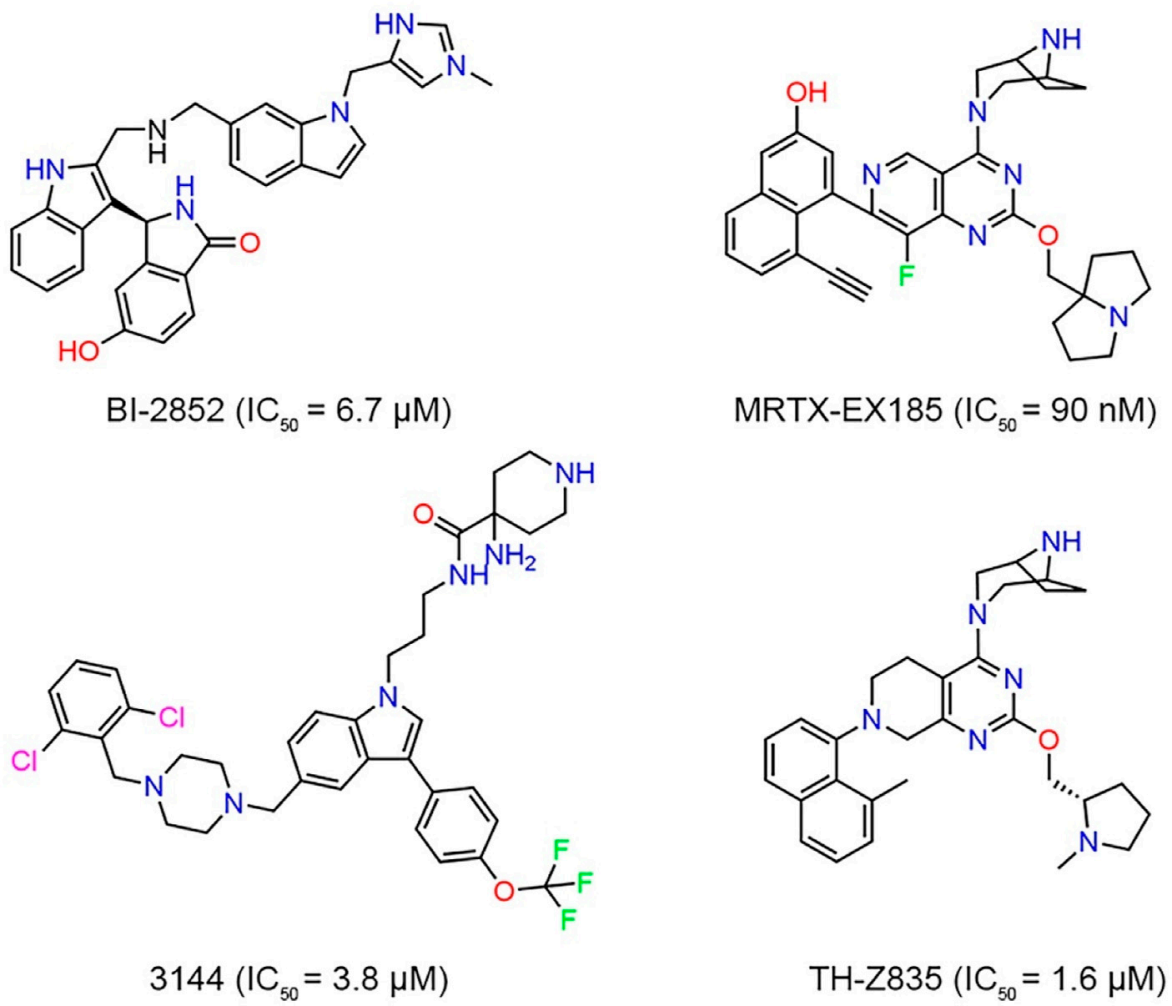
The chemical structures and biological data of known inhibitors of KRAS^G12D^.

Although KRAS^G12D^ is an excellent drug discovery target for many cancers, no drug directly targeting KRAS^G12D^ has been clinically approved. Targeting the KRAS^G12D^ mutation with a small molecule still remains a challenge due to lack of druggable pockets on the surface of RAS (Cox et al., 2014; Simanshu et al., 2017). However, with the study of RAS binding to low-molecular-weight organic molecules and the recent FDA approval of AMG 510 (Lumakras), a KRAS^G12C^ inhibitor binding to GDP, there has been a resurgence of research surrounding RAS (Chen et al., 2020; Lanman et al., 2020). Investigators reported a structural-designed KRAS inhibitor (BI-2852) that bound to the shallow pocket between switches I and II of KRAS^G12D^ and inhibited the protein-protein interaction between GDP-bound KRAS and SOS (Figure 1). However, limited cellular activity was observed (Kessler et al., 2019; Chen et al., 2020). Vasta et al. (2022) used the NanoBiT protein-protein interaction assay to evaluate the inhibitory effect of MRTX-EX185 on KRAS^G12D^ in cells and demonstrated that MRTX-EX185 is a potent KRAS^G12D^ inhibitor, albeit with low affinity. Welsch et al. designed a small molecule compound, named 3144, had affinity in the micromolar range, but toxicity and off-target activity of compound 3144 were detected in cells and mice, in addition, its low water solubility made it difficult to use in some contexts (Welsch et al., 2017; Khan et al., 2020). TH-Z835 was designed based on salt-bridge and induced fit pocket formation for KRAS^G12D^ targeting and inhibited the proliferation of cancer cells and significantly reduced the tumor volume. However, THZ835 had an off-target effect because the inhibition was not fully dependent on KRAS mutation status (Mao et al., 2022).

At present, the drug molecules with KRAS^G12D^ protein inhibitory activity reported have made slow progress in research and development (He et al., 2022). The novel molecular skeleton can provide more possibilities for specific effects or clinical studies, so it is necessary to study the new molecular skeleton of KRAS^G12D^ inhibitors (Peng et al., 2018). Molecular modeling of compound databases and virtual screening based on docking are one of the effective technologies to discover new chemical bioactive small molecules, which have been frequently used in the drug development of various targets (Ma et al., 2011). In this study, the primary objective was to discover KRAS^G12D^ inhibitors through structure-based pharmacophore modeling and virtual screening. First, a pharmacophore model based on KRAS^G12D^ crystal structure was constructed and used as a three-dimensional (3D) search query to retrieve potential inhibitors from commercial databases. Then the recovered hit compounds were filtered through molecular docking research to refine the hits. Finally, the four screened compounds showed sub-nanomolar affinities for KRAS^G12D^ and had a dose-dependent inhibitory effect on human pancreatic cancer cells. Among them, the hit compound 3 was the most promising compound with a significant inhibitory effect on tumor growth in mice.

## 2 Materials and methods

### 2.1 Materials

Human pancreatic cancer cell line (Panc 04.03) was obtained from American Type Culture Collection (ATCC) (Manassas, VA, United States) and cultured in Dulbecco’s modified Eagle’s medium (DMEM) with 1% penicillin-streptomycin and 10% fetal bovine serum (FBS) in a humidified atmosphere, 5% CO_2_ at 37°C. KRAS^G12D^ was purchased from Abcam (Cambridge, MA, United States). Hit compounds were purchased from WuXi AppTec.

### 2.2 Pharmacophore model generation

The virtual screening (VS.) method is designed to search large libraries of compounds *in silico*. The hit rate is usually much higher than traditional high throughput screening (HTS) (Tang et al., 2006; Shekhar, 2008). The crystal structure of KRAS^G12D^ (PDB ID: 7EWB) with a high resolution of 1.99 Å was downloaded from the Protein Data Bank (PDB) database and pretreated with the molecular operating environment (MOE) (Scholz et al., 2015) by removing water molecules, adding hydrogen atoms, and optimizing the orientation of hydrogen atoms. Based on the analysis of the interaction between the ligand and the amino acids in the KRAS^G12D^ active site, pharmacophore features were manually added using the pharmacophore editor of MOE (Nevin et al., 2012).

### 2.3 Virtual screening approach

An in-house chemical database contains 35,000 compounds with diverse structures and covers most of the chemical space, which is conducive to finding new skeleton molecules. The two-dimensional (2D) chemical structures of molecules in the database were firstly converted to 3D structures using the energy minimization algorithm of MOE software (MMFF94x force field) (Zheng et al., 2021). Through the pharmacophore search tool of the MOE (Zhang et al., 2020), all the compounds in the database were then screened according to the pharmacophore model, and the best-match hit compounds were evaluated using the root mean square distance (RMSD) values (The lower the RMSD values, the better the match). Finally, the hit compounds with an RMSD value of less than 0.09 Å were filtered by the molecular docking and the pharmacokinetic (PK) properties.

### 2.4 Molecular docking

Molecular docking was carried out through MOE software to evaluate the interaction between the hits of virtual screening and the active sites of KRAS^G12D^ (Scarpino et al., 2018). The crystal structure file of KRAS^G12D^ (PDB-ID:7EWB) was prepared for the docking studies where: 1) hydrogen atoms were added and water molecules were removed; 2) the partial charges were computed using Amber99 force field. The bound inhibitor TH-Z835 was used as the template to define the docking-active site in the crystal structure of KRAS^G12D^. Molecular docking was performed in the active site using the triangle matcher algorithm. Docking scores were calculated through the dG scoring function of MOE software. The lower docking score indicated better binding affinity between KRAS^G12D^ and ligand (Zheng et al., 2021).

### 2.5 In silico pharmacokinetic studies

In silico prediction of the pharmacokinetic (PK) properties of the hit compounds was performed by the ADMETlab tool (Mohammed, 2021). In these calculations, using reference 3144, we evaluated the common PK parameters such as the molecular weight (mol_MW ≤ 800), number of hydrogen bond donors (nHD ≤ 5), number of hydrogen bond acceptors (nHA ≤ 10), number of rotatable bonds (nRot ≤ 10), the aqueous solubility (−7 ≤ logS ≤ 0.5), topological polar surface area (TPSA ≤ 140), caco-2 permeability (CP ≥ -5.15), MDCK permeability, human intestinal absorption (HIA), F_20%_, F_30%_, volume of distribution (L/kg), T_1/2_, rat oral acute toxicity, eye corrosion, and eye irritation.

### 2.6 Microscale thermophoresis (MST) experiments

MST is an effective method to evaluate biomolecular interactions and has been used to study the interactions between binding partners of different molecular sizes (Wienken et al., 2010; Seidel et al., 2013). According to the previously reported method to measure the interaction between small molecule inhibitors and KRAS^G12D^ protein, the MST assay was performed using Monolith NT.115 instrument (NanoTemp Technologies, GmbH, Munich, Germany) (Wienken et al., 2010). The red fluorescent dye was used for KRAS^G12D^ fluorescent labeling. Small molecule inhibitors were diluted 1:1 and titrated. The binding buffer (50 mM Tris, 230 mM NaCl) was added to the dilution curve of the small molecule inhibitors and the labeled protein. The final sample was centrifuged at 13,000 rpm for 10 min, and then filled into the capillary tube for MST analysis with 20% LED power and 50% MST power at room temperature. The equilibrium dissociation constant (*K*_d_) was calculated using Nano Temper Analysis software.

### 2.7 MTT cell proliferation assay

According to the previously reported method (Yang et al., 2019), the human pancreatic cancer cell line (Panc 04.03) was seeded at the density of 2.4 × 10^4^ on 96-well plates. After incubation at 37°C with 5% CO_2_ for 24 h, the cells were incubated with different concentrations of inhibitors under the same conditions. After 72 h, MTT (0.5 mg/ml, 100 μl) was added after the supernatant was discarded. Then keep the 96-well plate at 37°C for 4 h. After that, abandon the medium and inject 200 μl of dimethyl sulfoxide (DMSO) into each well. The spectrophotometric absorbance of the sample at 570 nm was measured using a Synergy 4 Microplate Reader (BioTek Instruments Inc., United States). All of the compounds were tested three times in each of the cells.

### 2.8 *In vivo* anticancer activity

Based on previously reported protocols for investigating inhibition *in vivo* (Zhou et al., 2022), we injected Panc 04.03 cells (200 μl, 1 × 10^7^ cells) in the subdermal space on the right flank of 6-week-old BALB/c nude mice (Changzhou Cavens Laboratory Animal Co., Ltd, China). All experimental protocols were approved by the Animal Ethics Committee of China Pharmaceutical University (Ethic approval number: 2022–11–001). Once tumors grew to 80–100 mm^3^, mice were randomly divided into three groups (5 mice per group) and intraperitoneally administered with vehicle, hit compound 3 (3 mg/kg) and hit compound 3 (10 mg/kg) every 4 days for a total of five times. Tumor volume and body weight were measured every 4 days. Tumor volume was calculated using the formula (c × c × d)/2 (c, the smallest diameter; d, the largest diameter).

## 3 Results

### 3.1 Generation of pharmacophore model

**FIGURE 2:**
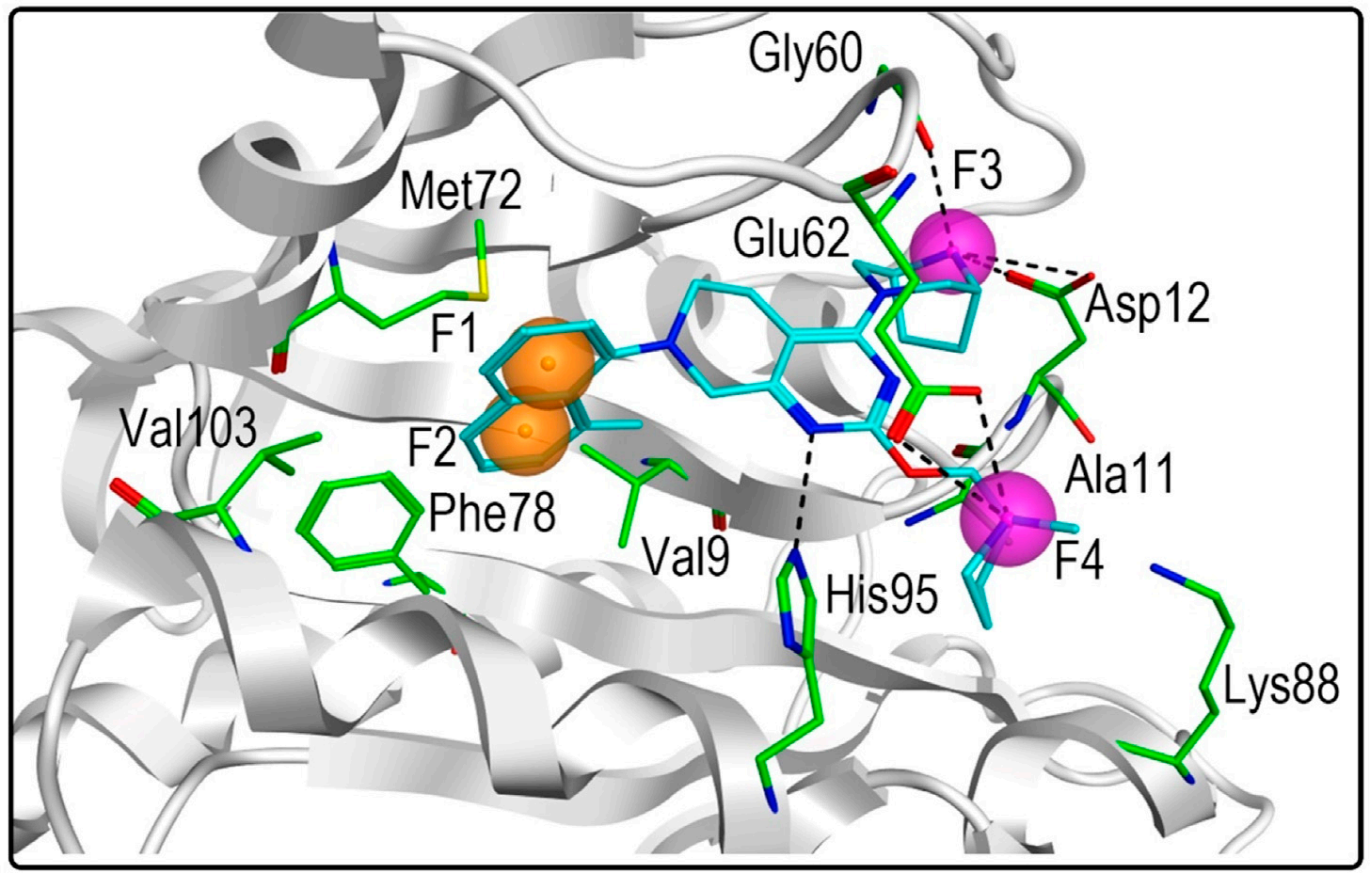
Pharmacophore model of KRAS^G12D^ was derived from the interaction of TH-Z835 and active-site amino acids. The ligand TH-Z835 is shown as cyan stick. Orange spheres correspond to aromatic features (F1 and F2: Aro), and purple spheres represent hydrogen-bond donors (F3 and F4: Don). Active-site residues are shown as green sticks. Hydrogen bonds are represented by black dotted lines.

The high-resolution X-ray structure of KRAS^G12D^ (PDB ID: 7EWB) in complex with the ligand TH-Z835 was downloaded from the PDB database. The generated structure-based pharmacophore model included four features (Figure 2): two aromatic features (F1 and F2: Aro) and two hydrogen-bond donor features (F3 and F4: Don). The two aromatic features of the pharmacophore model could be mapped onto the naphthalene ring of TH-Z835, which formed hydrophobic interactions with hydrophobic amino acids including Phe78, Met72, Val9 and Ala11. In addition, its hydrogen-bond donor features could match the nitrogen atoms of TH-Z835 that showed multiple hydrogen-bond interactions with Gly60, Glu62, Asp12, and His95. The above results reveal that this model may predict the spatial pharmacophore features of KRAS^G12D^ inhibitors.

### 3.2 Virtual screening

**FIGURE 3:**
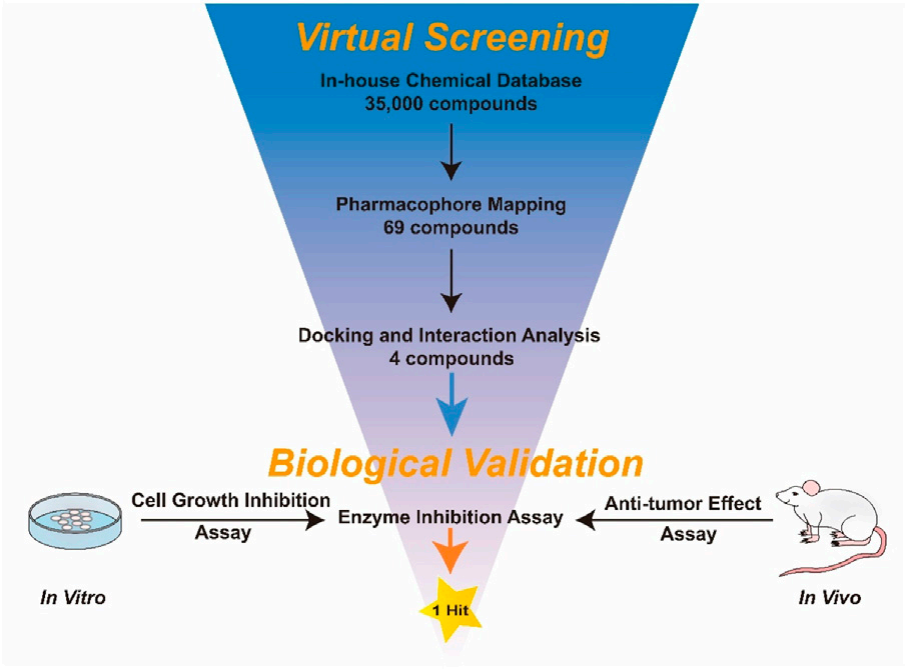
Schematic diagram of virtual screening and biological evaluation adopted in this study.

**FIGURE 4:**
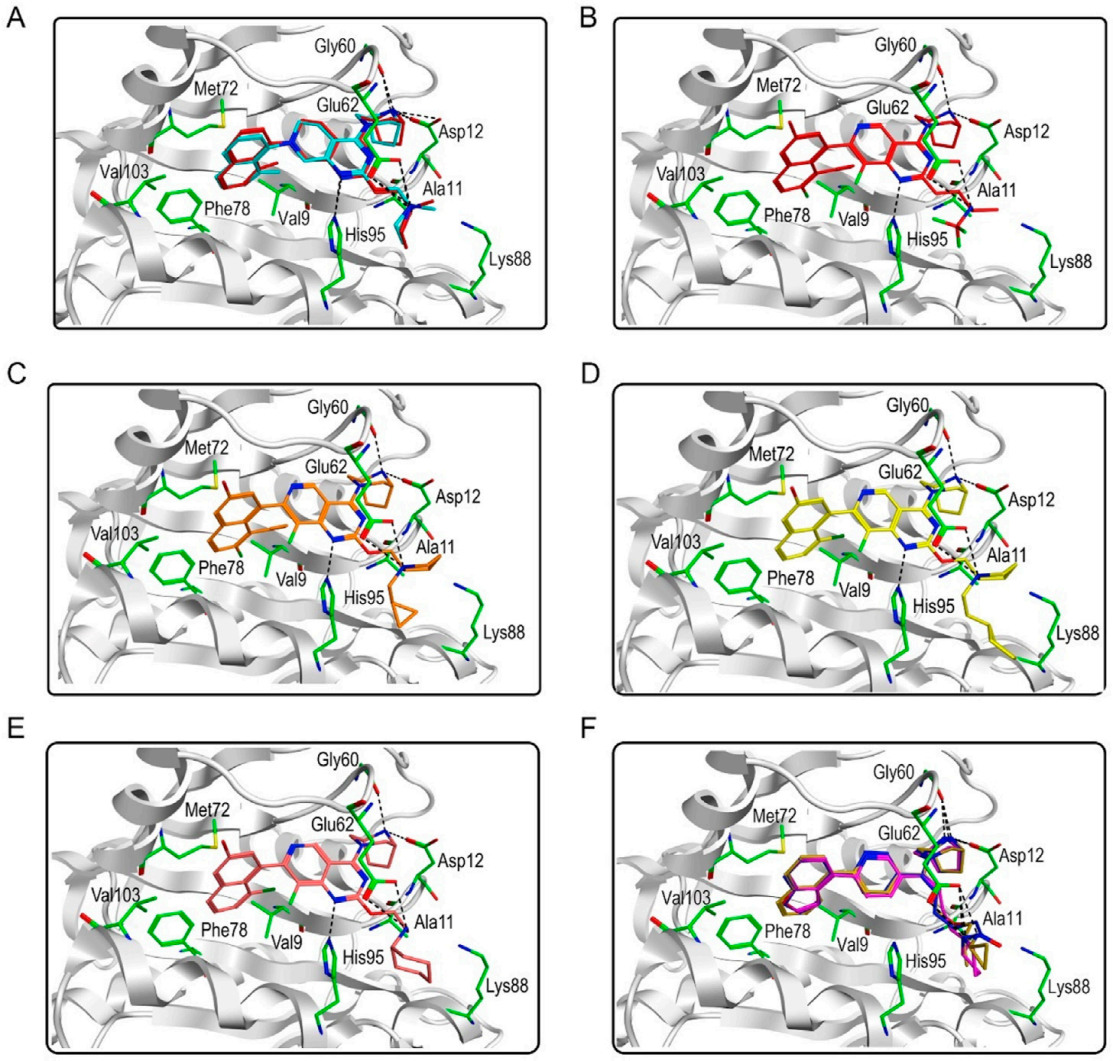
Predicted binding modes. **(A)** The docking conformation (red) and the actual conformation (cyan) of the co-crystallized ligand TH-Z835; **(B)** Hit compound 1; **(C)** Hit compound 2; **(D)** Hit compound 3; **(E)** Hit compound 4; **(F)** Hit compounds 5–7.

Figure 3 showed the virtual screen scheme that has been successfully used to identify inhibitors in other enzyme systems like Poly (ADP-Ribose) Polymerase-1 (PARP-1) (Zhou et al., 2019) and Tubulin (Zheng et al., 2021). MOE software was used for each step of virtual screening and molecular docking. Firstly, 35,000 compounds in the in-house chemical database were preliminarily screened according to the generated pharmacophore model of KRAS^G12D^. Then, 69 compounds with an RMSD value of less than 0.09 Å were selected for the docking-base screening. Before molecular docking, it is very necessary to verify the reliability of docking. In this study, we used the ligand (TH-Z835) of the crystal structure KRAS^G12D^-TH-Z835 complex (PDB ID: 7 EW B) as the template for verification. The co-crystallized ligand TH-Z835 was re-docked in the active site of KRAS^G12D^ (Figure 4A). We can observe that the docking conformation of TH-Z835 was well mapped with the actual conformation in the active site, indicating the good reliability of the docking method. Thus, this verified docking method can be used in virtual screening.

**FIGURE 5:**
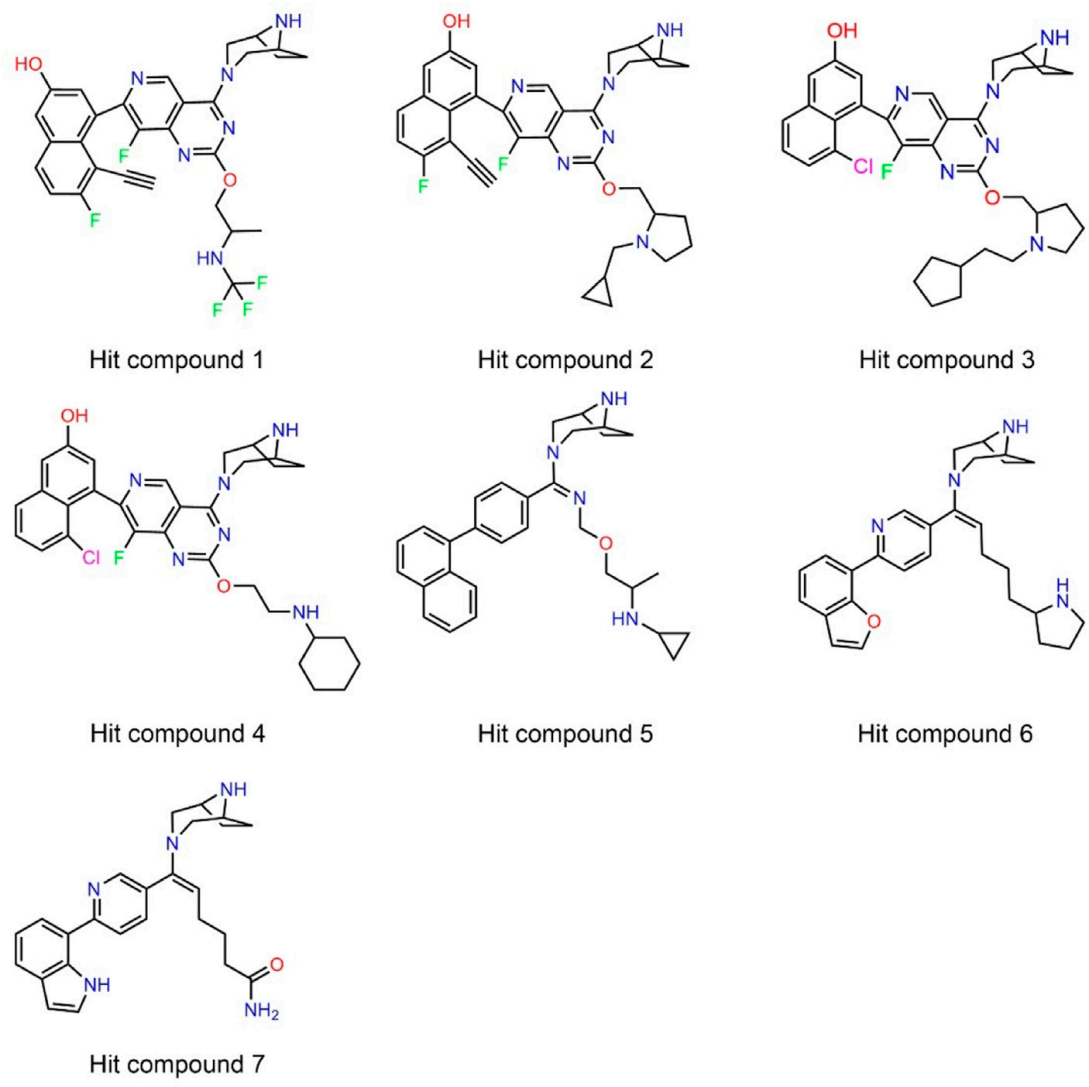
The chemical structures of hit compounds 1–7.

**FIGURE 6:**
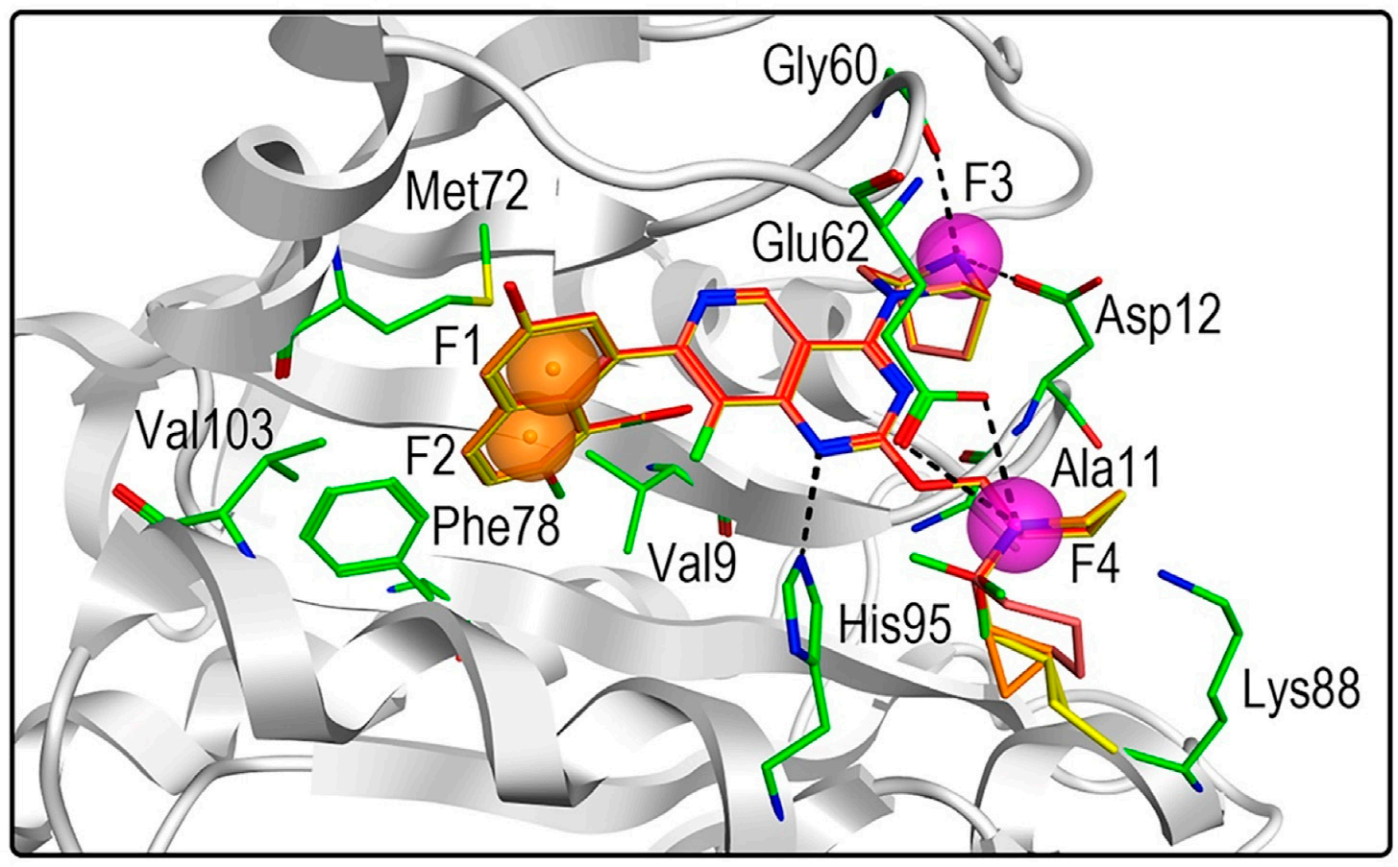
Three-dimensional pharmacophore mapping of hit compounds 1–4 with the active-site residues in KRAS^G12D^. Orange spheres correspond to aromatic features (F1 and F2: Aro), and purple spheres represent hydrogen-bond donors (F3 and F4: Don).

Next, 69 selected compounds of pharmacophore-base screening were docked into the KRAS^G12D^ binding site. TH-Z835 was used as the positive control and has a docking score of −10.04 kcal/mol. Based on docking scores, four top compounds (namely, hit compounds 1–4) below −10.04 kcal/mol and other three hit compounds (namely, hit compounds 5–7) ranking below them were further selected for predicting the three-dimensional interaction modes. Similar to TH-Z835, hit compounds 1–4 exhibited hydrogen-bond interactions with the key amino acids in the active site including His95, Glu62, Gly60, and Asp12 (Figures 4B–E). However, other hit compounds 5–7 could not form a hydrogen-bond interaction with His95 (Figure 4F). Compared with TH-Z835, halogen atoms in the skeleton of hit compounds 1–4 not only could exhibit stronger hydrophobic interactions with Val9, but also could greatly increase molecular liposolubility and metabolic stability. The chemical structures of all the hit compounds 1–7 were shown in Figure 5. In addition, a good pharmacophore mapping with the hit compounds 1–4 on the model was shown in Figure 6. The naphthalene ring of each hit could match the aromatic features of F1 and F2, while its nitrogen atoms were mapped onto the hydrogen-bond donor features of F3 and F4 respectively.

To provide a reliable pharmacokinetic (PK) property of a compound, *in silico* prediction of the PK properties of these four hit compounds were carried out using the reference inhibitor 3144 as reference (Table 1). In this calculation, we evaluated these common PK parameters including the molecular weight (mol_MW ≤ 800), number of hydrogen bond donors (nHD ≤ 5), number of hydrogen bond acceptors (nHA ≤ 10), number of rotatable bonds (nRot ≤ 10), the aqueous solubility (−7 ≤ logS ≤ 0.5), topological polar surface area (TPSA ≤ 140), caco-2 permeability (CP ≥ −5.15), MDCK permeability, human intestinal absorption (HIA), F_20%_, F_30%_, volume of distribution (L/kg), T_1/2_, rat oral acute toxicity, eye corrosion, and eye irritation. The result indicated that these parameter values of the hit compounds 1–4 are in the appropriate range. However, the reference inhibitor 3144 exceeded the specified range of nRot and caco-2 permeability respectively. Therefore, based on the molecular interaction and PK analysis, the four candidate hits (hit compounds 1–4) were finally chosen for further biological evaluation.

**TABLE 1:** Pharmacokinetic profile of the four hit compounds and reference 3144.

Profile	Compounds				
	Hit compound 1	Hit compound 2	Hit compound 3	Hit compound 4	3144
Molecular Weight (MW)	584.200	596.270	630.290	576.240	716.260
nHA	8	8	8	8	8
nHD	3	2	2	3	4
nRot	7	7	8	7	13
TPSA	95.430	86.640	86.640	95.430	87.790
logS	−6.016	−5.298	−5.329	−6.154	−4.822
Caco-2 Permeability (log unit)	−4.994	−4.906	−5.036	−5.124	−5.716
MDCK Permeability	1.7e-05	9.8e-06	1.2e-05	1.5e-05	5.1e-06
HIA	—	—	—	—	—
F_20%_	—	—	—	—	—
F_30%_	—	—	—	—	—
Volume of distribution (L/kg)	2.424	2.525	2.316	2.182	3.454
T_1/2_	.024	.015	.013	.020	.005
Rat Oral Acute Toxicity	—	—	—	—	—
Eye Corrosion	—	—	—	—	—
Eye Irritation	—	—	—	—	—

### 3.3 MST

Microscale Thermophoresis (MST) is a general method for quantifying binding affinity by measuring the equilibrium dissociation constant (*K*_d_) (Bartoschik et al., 2018). The MST binding assays showed that hits 1–4 effectively bound to the KRAS^G12D^ in the sub-nanomolar range (*K*_d_ = 0.13–0.98 nM) and displayed a stronger binding affinity for KRAS^G12D^ compared with the positive control TH-Z835 (Table 2).

**TABLE 2:** The docking scores and biological data of four hits.

Name	Docking score [kcal/mol]	*K*_d_ (nM)	IC_50_ (nM)^a^
Hit compound 1	−11.56	0.98 ± .09	212.23
Hit compound 2	−11.69	0.46 ± .08	89.96
Hit compound 3	−11.75	0.13 ± .05	43.80
Hit compound 4	−11.61	0.76 ± .11	145.97
TH-Z835	−10.04	1.08 ± 0.07 µM	>300

a IC_50_ (nM) is the concentration of compound needed to inhibit cell growth by 50% following 72 h cell treatment with hit compounds 1-4 and TH-Z835, respectively.

### 3.4 Cell growth inhibitory activity

**FIGURE 7:**
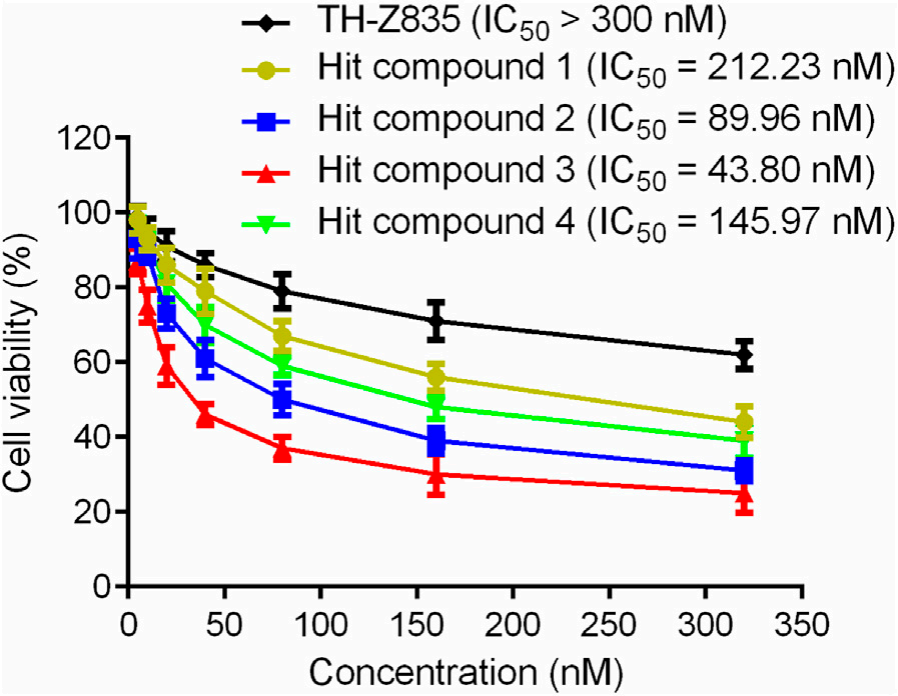
Growth inhibition effects of hit compounds 1–4 and TH-Z835 on Panc 04.03 cells. Results are expressed as mean ± SD, *n* = 3.

In this experiment, the MTT method was used to detect the effects of hit compounds 1–4 s on the proliferation of human pancreatic cancer cells (Panc 04.03). The results showed that hits 1–4 had a dose-dependent effect on the proliferation of Panc 04.03 cells (Figure 7). In addition, their IC_50_ values were further calculated. Compared with other hits and the positive control TH-Z835, hit 3 with the IC_50_ value of 43.80 nM exhibited a stronger anti-proliferative activity on Panc 04.03 cells. Therefore, hit compound 3 was selected as a lead compound and further used for anti-tumor experimental evaluation *in vivo*.

### 3.5 Tumor growth inhibition *in vivo*

**FIGURE 8:**
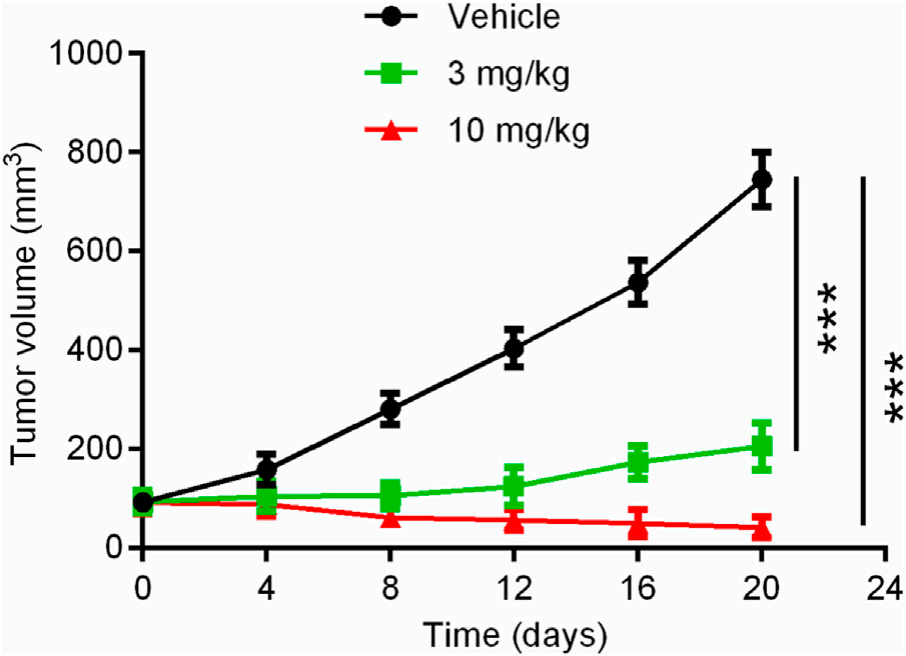
Mean tumor volume as a function of time (days) after the treatment of the hit compound 3. Data are represented as mean ± SD, *n* = 5. ***p < .001.

Given the significant inhibitory effect of hit compound 3 on pancreatic cancer cell growth *in vitro*, the anti-pancreatic cancer activity of hit compound 3 was evaluated. The mice were randomly divided into three groups (5 mice per group): the vehicle control group, hit compound 3 (3 mg/kg) treated group and hit compound 3 (10 mg/kg) treated group. The hit compound 3 significantly inhibited the growth of Panc 04.03 in a concentration-dependent manner after intraperitoneal injection for 20 days. As shown in Figure 8, the growth rate of xenograft tumors in BALB/c nude mice after hit compound 3 administration was significantly reduced compared with the untreated group. In addition, the significant difference in tumor volume between the untreated and treated groups further demonstrated the inhibitory effect of hit compound 3 on xenograft tumor growth in BALB/c nude mice *in vivo*.

## 4 Discussion and conclusion

In order to develop inhibitors of KRAS^G12D^, the most common KRAS mutant in pancreatic cancer, we used a structure-based screening method to successfully identify four hit compounds and evaluated their activity *in vitro* and *in vivo*. The MST experimental result showed that hits 1–4 had sub-nanomolar affinities for KRAS^G12D^. Particularly, hit compound 3 had a remarkable anti-proliferation effect on human pancreatic cancer cells and significantly inhibited tumor growth in tumor-bearing mice. Since hit compound 3 is the most promising lead compound targeting KRAS^G12D^, structural optimization of hit compound 3 is currently under way in our laboratory to further explore structurally novel and more effective KRAS^G12D^ inhibitors. In addition, the results of this experiment prove that this screening scheme provides guidance in identifying potent KRAS^G12D^ inhibitors, and may be applicable to other KRAS family members in the future.

## Data Availability

The original contributions presented in the study are included in the article/Supplementary Material, further inquiries can be directed to the corresponding authors.
